# Downregulation of T-cell cytotoxic marker IL18R1 promotes cancer proliferation and migration and is associated with dismal prognosis and immunity in lung squamous cell carcinoma

**DOI:** 10.3389/fimmu.2022.986447

**Published:** 2022-12-05

**Authors:** Qiang Guo, Chuang-Yan Wu, Ni Jiang, Song Tong, Jun-Hao Wan, Xiao-Yue Xiao, Pei-Yuan Mei, Hua-Song Liu, Si-Hua Wang

**Affiliations:** ^1^ Department of Thoracic Surgery, Union Hospital, Tongji Medical College, Huazhong University of Science and Technology, Wuhan, China; ^2^ Department of Obstetrics and Gynecology, Women and Children’s Hospital of Chongqing Medical University, Chongqing, China; ^3^ Department of Cardiothoracic Surgery, Taihe Hospital, Hubei University of Medicine, Shiyan, China

**Keywords:** interleukin 18 receptor 1, lung squamous cell carcinoma, immune microenvironment, T cytotoxic cells, prognosis

## Abstract

Immunotherapy can improve the survival of patients with advanced lung squamous cell carcinoma (LUSC). T cytotoxic cells are one of the main members of the immune microenvironment. Herein, we aimed to identify the roles of T-cell cytotoxic markers interleukin 18 (IL18) receptor 1 (IL18R1) in the LUSC progression using bioinformatics, clinical tissue specimen, and cell experiment. We assessed the association between the IL18R1 expression and immune infiltration and IL18R1-related competing RNA network. The IL18R1 expression was downregulated in the LUSC tissues. The IL18R1 expression downregulation was associated with diagnosis and short overall survival and disease-specific survival, and it was also an independent risk factor for dismal survival time in LUSC. IL18R1-related nomograms predicted the survival time of patients with LUSC. IL18R1 overexpression inhibited the proliferation, migration, and invasion of LUSC cells. The IL18R1 expression was significantly associated with the microenvironment (stromal, immune, and estimate scores), immune cells (such as the T cells, cytotoxic cells, CD8 T cells), and immune cell markers (such as the CD8A, PD-1, and CTLA4) in LUSC. AC091563.1 and RBPMS-AS1 downregulation was positively associated with the IL18R1 expression, negatively associated with the miR-128-3p expression, and associated with short disease-specific survival and progression in LUSC. In conclusion, IL18R1 was significantly downregulated and associated with the prognosis and immune microenvironment. IL18R1 overexpression inhibits the growth and migration of cancer cells in LUSC. Furthermore, AC091563.1 and RBPMS-AS1 might compete with IL18R1 to bind miR-128-3p for participating in LUSC progression. These results showed that IL18R1 is a biomarker for evaluating the prognosis of patients with LUSC.

## Introduction

Immunotherapy induces changes in the immune microenvironment of patients with lung squamous cell carcinoma (LUSC) during the courses of immunotherapy. The immune microenvironment is closely associated with cancer progression (1–3). Jin et al. reported that the tumor immune microenvironment heterogeneity is related to oncogenic patterns. Furthermore, PD-L1 and TIL positivity are significantly associated with KRAS mutations. PD-L1 and TIL are elevated in patients with EGFR mutations or ALK rearrangements. The EGFR L858R mutation is positively associated with inflammatory phenotype, and the anti-PD-1/PD-L1 therapy is effective (2). Tumors can affect the tumor microenvironment *via* cell-signaling molecules to promote tumorigenesis and development. Moreover, the immune microenvironment consists of immune cells, fibroblasts, bone marrow-derived inflammatory cells, and other cells. T cytotoxic cell is one of the main members of the immune microenvironment and plays an important role in tumor progression (4, 5).

Interleukin 18 (IL18) receptor 1 (IL18R1), also known as IL18RA, encodes a cytokine receptor. The receptor encoded by IL18R1 specifically binds to IL18, thereby exerting biological effects. T cytotoxic cells are known as CD8^+^ T cells. IL18R1 is the surface marker of CD8^+^ T cells (6). IL18R1 plays an important role in cancer progression (7–10). For example, IL18R1 is overexpressed in breast cancer (7). Furthermore, the IL18R1 expression level is positively associated with the disease-free survival (DFS) of triple-negative patients with breast cancer (8). Overexpression of the inflammatory cytokine IL18 is related to a dismal prognosis in patients with liver cancer. Moreover, IL18R1 deletion could enhance tumor growth and burden, and it increased the tumor burden in the carcinogen-induced liver cancer model. IL18 could exert an inflammation-dependent tumor suppressor effect by promoting tumor-infiltrating T-cell differentiation, activity, and survival (9). In comparison to anti-PD-1 therapy, TUSC2 and anti-PD-1 therapy can reduce tumor growth and prolong tumor survival. In subcutaneous tumors and lung metastases, a combined treatment of TUSC2 and anti-PD-1 promoted an increase in tumor-infiltrating NK and CD8 T cells, whereas the MDSC and Treg levels decreased. The IL18R1 expression level was associated with the activation of NK cells by TUSC2 (10). Targeted therapy and immunotherapy have recently become hotspots in cancer research. However, patients with LUSC lacked effective targets to improve patient prognosis. At present, the roles of the T cytotoxic cell surface marker IL18R1 in LUSC have not been reported. Therefore, we aimed to investigate the functions of IL18R1 in LUSC progression and the association between the immune microenvironment and IL18R1 expression to identify patients with cancer who would benefit from the prognosis.

## Materials and methods

### Identification of IL18R1 expression levels in LUSC tissues

We identified the IL18R1 expression level using the RNA data of 49 normal lung and 502 LUSC tissues derived from the Cancer Genome Atlas (TCGA) website and 288 normal lung tissues derived from Genotype-Tissue Expression (GTEx) website (11, 12). The data included the two (TPM and FPKM) types. In the lung cancer explorer (LCE) database (13), the IL18R1 expression level in the LUSC tissues was investigated *via* a meta-analysis of data obtained from the TCGA and gene expression omnibus (GEO) databases. We collected cancer and normal tissue samples from patients with LUSC at Wuhan Union Hospital, China. These specimens were all approved by the ethical committee of our hospital, and all patients provided informed consent. There were 9 normal lung tissue samples and 32 LUSC tissue samples.

### Immunohistochemistry

We obtained the tissue sections from the Pathology Department of our hospital and routinely dewaxed them. After washing, nonspecific background staining was reduced and antigen repair was done. After buffer washing, we incubated the tissue sections overnight with a drop of 1:200 IL18R1 antibody (Bioss, China) working solution. The next day, the tissue sections were stained, counterstained, dehydrated, transparent, and sealed. Next, we took photos. The IL18R1 expression levels in normal and LUSC tissues were analyzed using a scanner and Quant Center 2.3 software (14).

### Roles of IL18R1 expression in the diagnosis of LUSC

We identified the roles of IL18R1 expression in the diagnosis of LUSC *via* diagnostic analysis using the RNA data derived from the TCGA and GTEx databases (15). The diagnostic data type included the TPM and FPKM types, and the area under the curve (AUC) was used as an evaluation criterion for diagnostic value.

### Association of IL18R1 expression in assessing prognosis in patients

LUSC patients were divided into high- and low-IL18R1 expression groups based on the median value of IL18R1 expression. The association between IL18R1 expression level and overall survival (OS), disease-specific survival (DSS), and progress-free interval (PFI) of patients with LUSC were investigated *via* the Kaplan-Meier (K-M) survival analysis using the TCGA database. The grouping criterion was the median value of IL18R1 expression. In the LCE database, the association between IL18R1 level and the OS of LUSC patients was studied by meta-analysis using the data of patients with LUSC from the TCGA and GEO databases (13).

### Construction of IL18R1-related prognostic nomogram

Univariate COX regression analysis revealed associations between the TNM stage, pathological stage, sex, age, smoking history, and IL18R1 expression and OS, PFI, and DSS in patients with LUSC. Multivariate COX regression analysis indicators were screened with a P < 0.05, and an IL18R1-related prognostic nomogram was constructed based on the multivariate COX regression analysis results (16).

### Construction of cell model

The SK-MES-1 cells were purchased from PRICELLA, Wuhan city, and were fed with the 10% fetal bovine serum. The vector (pcDNA3.1(+)) was constructed at GENERAL BIOL (China), and the IL18R1 overexpression plasmid was synthesized. In the condition of a good growth state, DNA plasmid and transfection reagent lipofectamine2000 were diluted in the Opti-MEM (reduced-serum medium). The diluted DNA plasmid and lipofectamine2000 were mixed and incubated for 20 min at room temperature to add to the medium containing SK-MES-1 cells. The medium was changed 6 h after transfection, and the IL18R1 expression levels in SK-MES-1 cells of the two groups were detected using quantitative real-time polymerase chain reaction (PCR) and western blotting (WB).

### Identification of cell model

The LUSC cells of the negative control group and IL18R1 overexpression group were collected for extracting the total RNAs and proteins. The IL18R1 mRNA and protein expression levels in the IL18R1 overexpression cell model were detected according to the standard procedures of quantitative real-time PCR and WB (14). The PCR primer sequences were as follows: IL18R1 forward: CGCCGAGTTTGAAGATCAGG, reverse: TCAGCAAAGCAGAGCAGTTG, and GAPDH forward: TCAAGAAGGTGGTGAAGC AGG, reverse: TCAAAGGTGGAGGAGTGG GT. During incubation with WB, the antibody concentrations were 1:2000 IL18R1 antibody (Bioss, China) and 1:20000 GAPDH antibody (Servicebio, China), respectively.

### Cell proliferation

After digestion, centrifugation, resuspension, and counting, the model cells were incubated on 96-well plates, and the cell suspension was incubated at 100 μL/well at 37°C and 5% CO_2_. Next, 10 μL CCK-8 (Biosharp, China) was joined to each well at 0, 24, 48, and 72 h, and the absorbance values at 450 nm were measured using a microplate reader after co-culture for 2 h in the incubator.

### TUNEL experiment

The LUSC model cells were fixed with paraformaldehyde. The slides were washed with phosphate buffer solution (PBS) and 20 μg/mL DNase I free enzyme (PK) solution was added to droppers. PK was removed by using PBS after incubating for 5 min at room temperature. Terminal deoxynucleotidyl transferase was added and incubated for 1 h in the dark. The DAPI (4′,6-diamidino-2-phenylindole) solution was added after the slides were washed using PBS, and slide fixation was performed through a mounting solution containing an anti-fluorescence quencher. Finally, the images were taken under a fluorescence confocal microscope.

### Cell migration and invasion

A marker pen was used to evenly inscribe the back of the 6-well plates. Subsequently, the model cells were fed in those 6-well plates. The second day was scratched with the horizontal line marked by using the 200 μL vertical plate of the spearhead. The model cells were washed thrice with PBS to wash the exfoliated cancer cells, and serum-free medium was subsequently added to the 6-well plates. The LUSC cells were reared in an incubator at 37°Cand 5% CO_2_. We took photos at 0, 12, 24, 36, and 48 h for statistics. The matrigel gel was diluted, and coated with the bottom of a Transwell chamber. The starved cell suspensions were prepared, and 100 μL of the serum-free cell suspension was added to the Transwell chamber. Next, 600 μL of complete medium containing 10% fetal bovine serum was added to the lower chamber of 24-well plates. Transwell chambers were removed when the LUSC cells were reared at 37°C and 5% CO_2_ incubator for 24 h. Then, the chambers were fixed, stained, photographed, and counted.

### Association between IL18R1 and LUSC immune microenvironment

ESTIMATE and ssGSEA algorithms were used to calculate the expression levels of immune microenvironment components in LUSC tissues (17). The association between IL18R1 expression and stromal, immune, and estimate scores and 24 immune cells was visualized by Pearson analysis and scatter plot. The association between stromal, immune, and estimate scores and 24 immune cells in IL18R1 overexpression and IL18R1 low expression groups was investigated. The RNA data of cell markers were extracted from the TCGA LUSC tissues, and the association between the IL18R1 level and the levels of cell markers was analyzed *via* Pearson association analysis.

### Association between IL18R1 and the competitive RNA network

MicroRNAs (miRNAs) upstream of IL18R1 were predicted in the ENCORE database with cancer type greater than or equal to 1, and were identified in LUSC tissues, with elevated miRNAs expression levels and P < 0.05 as the filter criteria. Furthermore, IL18R1 negatively associated with miRNAs in the LUSC tissues was used to predict long non-coding RNAs (lncRNAs). In the ENCORI database, the lncRNAs bound upstream of miR-128-3p and miR-556-5p were predicted with cancer type greater than or equal to 6, with the filter criteria of P < 0.05 and overexpressed lncRNAs in LUSC tissues. Moreover, the negatively associated lncRNAs of miR-128-3p and miR-556-5p, the positively associated lncRNAs of IL18R1, and prognosis-related lncRNAs were used as the inclusion criteria to form a competitive RNAs (ceRNAs) network.

### Statistical analysis

The Wilcoxon rank sum test was applied to detect the IL18R1 levels in normal and LUSC tissues and assess whether immune cells had statistical significance in the IL18R1 expression groups. The IL18R1 expression levels in normal and LUSC tissues and the association between IL18R1 level and survival time of patients with LUSC were revealed using the meta-analysis. The association between IL18R1 expression level and immune microenvironment was obtained using correlation analysis. The t-test was used to determine whether the role of IL18R1 in cell growth and migration was statistically significant. P < 0.05 was considered statistically significant.

## Results

### Downregulation of IL18R1 expression level in LUSC tissues

**Figure 1:**
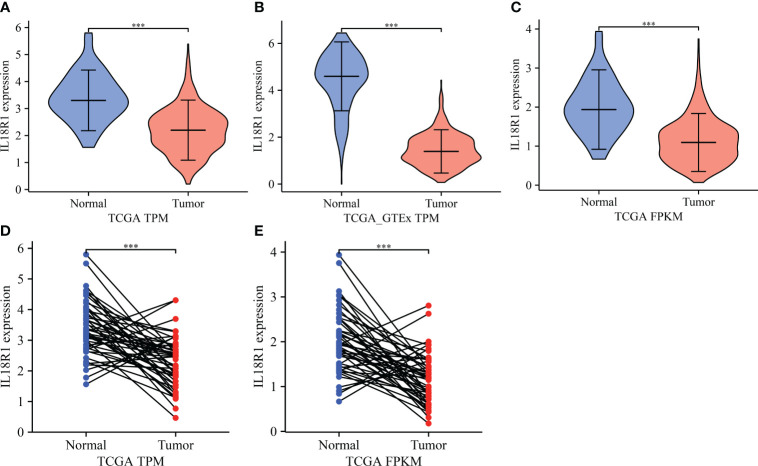
IL18R1 was significantly downregulated in the LUSC tissues based on the data of TPM and FPKM types in the TCGA and GTEx databases. **(A–C)** Unpaired tissues in LUSC; **(D, E)** Paired tissues in LUSC. LUSC, lung squamous cell carcinoma; TCGA, The Cancer Genome Atlas; GTEx, Genotype-Tissue Expression; ***P < 0.001.

**Figure 2:**
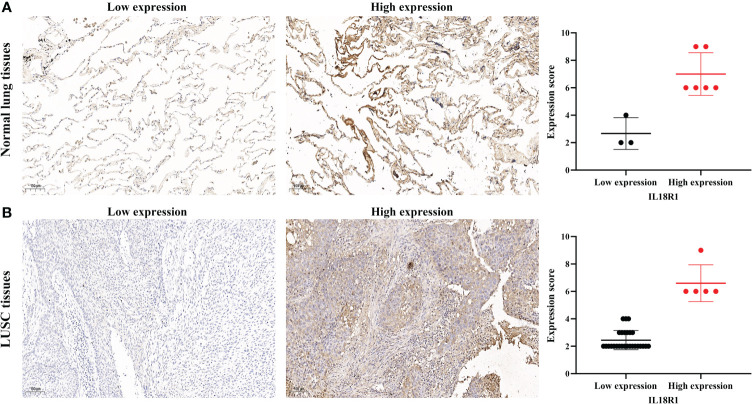
IL18R1 was significantly downregulated in the LUSC tissues of our hospital. **(A)** Normal lung tissues; **(B)** LUSC tissues. LUSC, lung squamous cell carcinoma.

Differential expression analysis revealed that the IL18R1 level in LUSC tissues was significantly reduced in the data of TPM type (**Figure 1A–C**). In patients with paired LUSC, IL18R1 was significantly underexpressed in the LUSC tissues of the TCGA database (**Figures 1D, E**). In addition, a meta-analysis based on LUSC data came in LCE database found that the IL18R1 expression level was significantly reduced in the LUSC tissues (**Figure S1**). In the clinical LUSC samples, the changes of IL18R1 level were consistent with that in the database analysis results. Among the nine normal tissue samples, the IL18R1 expression levels significantly increased in six (66.67%) normal tissue samples. Among the 32 LUSC tissue samples, five (15.625%) cancer tissue samples showed a significant increase in the IL18R1 expression levels (**Figure 2** and **Table 1**).

**Table 1:** The IL18R1 expression levels in the tissues of patients with LUSC.

Number	Proportion Score	Intensity Score	IRS score
Normal tissues	1	2	2
Normal tissues	1	2	2
Normal tissues	2	2	4
Normal tissues	2	3	6
Normal tissues	2	3	6
Normal tissues	2	3	6
Normal tissues	2	3	6
Normal tissues	3	3	9
Normal tissues	3	3	9
LUSC tissues	1	2	2
LUSC tissues	1	2	2
LUSC tissues	1	2	2
LUSC tissues	1	2	2
LUSC tissues	1	2	2
LUSC tissues	1	2	2
LUSC tissues	1	2	2
LUSC tissues	1	3	3
LUSC tissues	1	2	2
LUSC tissues	1	2	2
LUSC tissues	1	2	2
LUSC tissues	1	2	2
LUSC tissues	1	2	2
LUSC tissues	1	3	3
LUSC tissues	1	3	3
LUSC tissues	1	3	3
LUSC tissues	1	2	2
LUSC tissues	1	3	3
LUSC tissues	1	2	2
LUSC tissues	1	3	3
LUSC tissues	1	2	2
LUSC tissues	1	2	2
LUSC tissues	1	2	2
LUSC tissues	1	2	2
LUSC tissues	2	2	4
LUSC tissues	2	2	4
LUSC tissues	2	2	4
LUSC tissues	2	3	6
LUSC tissues	2	3	6
LUSC tissues	2	3	6
LUSC tissues	2	3	6
LUSC tissues	3	3	9

LUSC, lung squamous cell carcinoma.

### Association between decreased IL18R1 expression level and the diagnosis and dismal prognosis in LUSC

**Figure 3:**
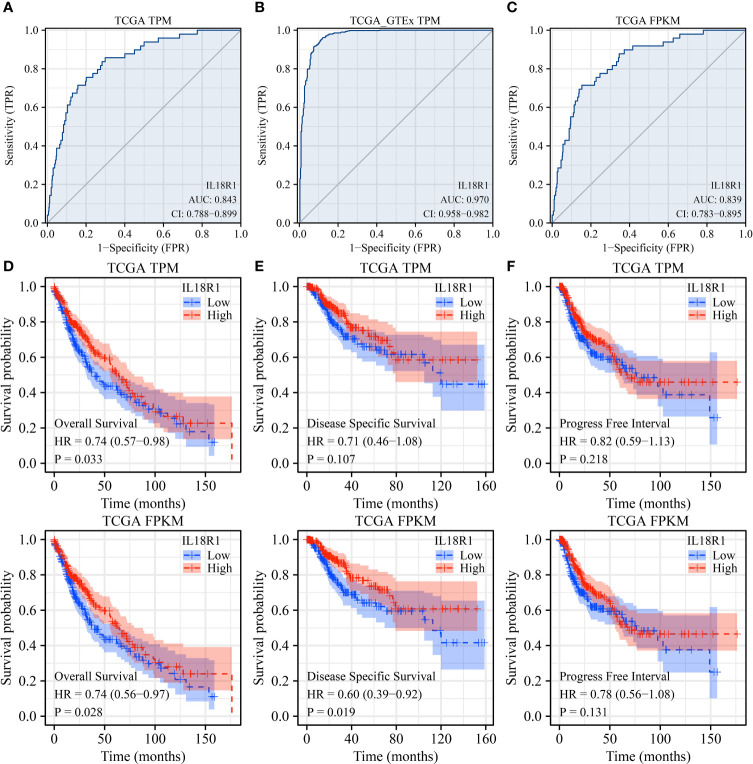
IL18R1 plays a role in the diagnosis and prognosis of patients with LUSC based on the data of TPM and FPKM types in the TCGA and GTEx databases. **(A–C)** The results of ROC analysis; **(D)** OS; **(E)** DSS; **(F)** PFI. Note: TCGA, The Cancer Genome Atlas; GTEx, Genotype-Tissue Expression; LUSC, lung squamous cell carcinoma; TCGA, The Cancer Genome Atlas; GTEx, Genotype-Tissue Expression; OS, overall survival; DSS, disease-specific survival; PFI, progress-free interval.

AUC was greater than 0.75 in the normal and cancer tissues using receiver operating characteristic (ROC) analysis. In detail, based on the data from the TCGA and TCGA-GTEx TPM types, the AUCs of IL18R1 in the diagnosis of LUSC were found to be 0.843 and 0.970, respectively (**Figure 3A, B**). Analysis of the TCGA FPKM type data revealed an AUC of 0.839 for IL18R1 in the diagnosis of LUSC (**Figure 3C**), suggesting that the IL18R1 expression levels helped in the diagnosis of LUSC. The K-M survival studies have found that the decreased IL18R1 level was relevant to the dismal prognosis in LUSC patients (**Figure 3D, E**). Specifically, the decreased IL18R1 levels were relevant to the shorter OS and DSS in LUSC patients. Meta-studies have shown that the decreased IL18R1 level was relevant to a shorter OS in patients with LUSC (**Figure S2**).

### IL18R1-related prognostic nomogram

**Figure 4:**
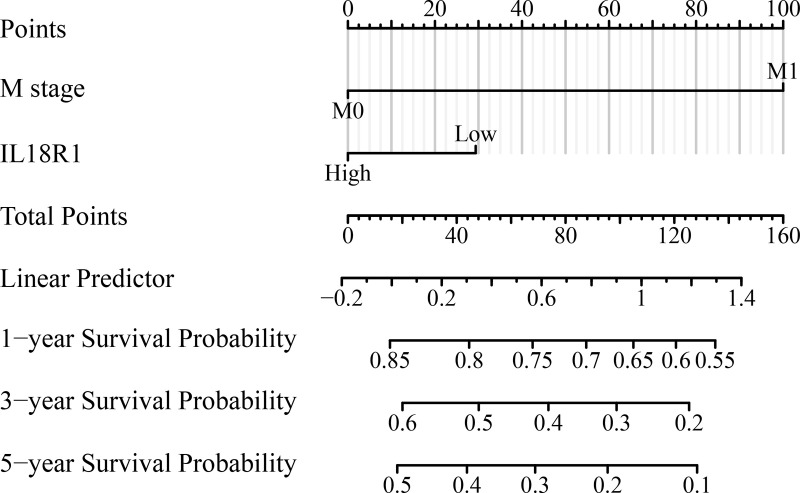
IL18R1-related prognostic nomogram.

Univariate COX studies revealed that T, M, and pathological stage, and IL18R1 level were the risk indicators for OS in patients with LUSC (**Table 2**). T, N, and pathological stage, and IL18R1 expression were the risk indicators for DSS in patients with LUSC (**Table 3**). T, and pathological stage, and smoking history were the risk indicators for PFI in patients with LUSC (**Table 4**). Multivariate COX studies revealed that the M stage and IL18R1 expression level were independent risk factors for OS in patients with LUSC (**Table 2**). The IL18R1 expression level was an independent risk factor for DSS in patients with LUSC (**Table 3**). A nomogram of IL18R1-related OS was established based on the results of COX studies (**Figure 4**).

**Table 2:** Overall survival factors in patients with LUSC.

Characteristics	Total (N)	HR (95% CI)	P	HR (95% CI)	P
T stage	496				
T1	114	Reference			
T2	289	1.237 (0.872-1.753)	0.233	1.185 (0.800-1.756)	0.398
T3	70	1.816 (1.160-2.843)	0.009	1.489 (0.817-2.716)	0.194
T4	23	2.324 (1.248-4.327)	0.008	1.822 (0.781-4.251)	0.165
N stage	490				
N0	317	Reference			
N1	128	1.076 (0.786-1.473)	0.646		
N2	40	1.327 (0.836-2.106)	0.230		
N3	5	2.525 (0.621-10.263)	0.195		
M stage	415				
M0	408	Reference			
M1	7	3.112 (1.272-7.616)	0.013	2.847 (1.106-7.333)	0.030
Pathologic stage	492				
Stage I	243	Reference			
Stage II	159	1.142 (0.830-1.572)	0.414	1.109 (0.766-1.604)	0.584
Stage III	83	1.561 (1.093-2.229)	0.014	1.123 (0.672-1.878)	0.658
Stage IV	7	3.325 (1.346-8.213)	0.009		
Gender	496				
Female	130	Reference			
Male	366	1.211 (0.879-1.669)	0.241		
Age	490				
<=65	190	Reference			
>65	300	1.279 (0.960-1.704)	0.093		
Smoker	484				
No	18	Reference			
Yes	466	0.585 (0.259-1.325)	0.199		
IL18R1	496				
Low	246	Reference			
High	250	0.738 (0.563-0.968)	0.028	0.702 (0.519-0.950)	0.022

LUSC, lung squamous cell carcinoma.

**Table 3:** Disease-specific survival factors in patients with LUSC.

Characteristics	Total (N)	HR (95% CI)	P	HR (95% CI)	P
T stage	444				
T1	106	Reference			
T2	255	1.335 (0.754-2.362)	0.322	1.190 (0.652-2.171)	0.570
T3	65	3.117 (1.623-5.989)	<0.001	2.613 (0.979-6.975)	0.055
T4	18	3.577 (1.305-9.809)	0.013	2.307 (0.655-8.132)	0.193
N stage	440				
N0	291	Reference			
N1	113	1.444 (0.896-2.328)	0.131	1.082 (0.512-2.288)	0.836
N2	32	2.576 (1.394-4.759)	0.003	1.498 (0.426-5.270)	0.529
N3	4	0.000 (0.000-Inf)	0.994	0.000 (0.000-Inf)	0.995
M stage	365				
M0	358	Reference			
M1	7	2.800 (0.680-11.522)	0.154		
Pathologic stage	440				
Stage I	221	Reference			
Stage II	143	1.600 (0.956-2.675)	0.073	1.191 (0.525-2.700)	0.676
Stage III	69	3.108 (1.840-5.250)	<0.001	1.467 (0.384-5.609)	0.575
Stage IV	7	3.635 (0.864-15.283)	0.078	2.595 (0.512-13.152)	0.250
Gender	444				
Female	113	Reference			
Male	331	1.386 (0.833-2.307)	0.209		
Age	438				
<=65	175	Reference			
>65	263	1.028 (0.668-1.582)	0.899		
Smoker	432				
No	16	Reference			
Yes	416	0.393 (0.123-1.251)	0.114		
IL18R1	444				
Low	221	Reference			
High	223	0.599 (0.390-0.920)	0.019	0.585 (0.375-0.911)	0.018

LUSC, lung squamous cell carcinoma.

**Table 4:** Progress-free interval factors in patients with LUSC.

Characteristics	Total (N)	HR (95% CI)	P	HR (95% CI)	P
T stage	497				
T1	114	Reference			
T2	290	1.209 (0.789-1.853)	0.384	1.085 (0.685-1.719)	0.727
T3	70	2.709 (1.641-4.473)	<0.001	1.860 (0.992-3.489)	0.053
T4	23	1.970 (0.862-4.503)	0.108	1.284 (0.470-3.507)	0.625
N stage	491				
N0	317	Reference			
N1	129	1.557 (1.091-2.222)	0.015		
N2	40	1.582 (0.924-2.708)	0.094		
N3	5	0.000 (0.000-Inf)	0.995		
M stage	416				
M0	409	Reference			
M1	7	1.709 (0.421-6.937)	0.453		
Pathologic stage	493				
Stage I	243	Reference			
Stage II	160	1.542 (1.056-2.251)	0.025	1.269 (0.827-1.947)	0.276
Stage III	83	2.241 (1.470-3.416)	<0.001	1.746 (1.018-2.996)	0.043
Stage IV	7	2.145 (0.522-8.814)	0.290	1.954 (0.459-8.316)	0.365
Gender	497				
Female	130	Reference			
Male	367	1.154 (0.789-1.688)	0.460		
Age	491				
<=65	191	Reference			
>65	300	1.123 (0.802-1.572)	0.500		
Smoker	485				
No	18	Reference			
Yes	467	0.424 (0.186-0.966)	0.041	0.471 (0.205-1.083)	0.076
IL18R1	497				
Low	247	Reference			
High	250	0.779 (0.564-1.077)	0.131		

LUSC, lung squamous cell carcinoma.

### Upregulation of IL18R1 expression inhibits the growth and metastasis of LUSC cells

**Figure 5:**
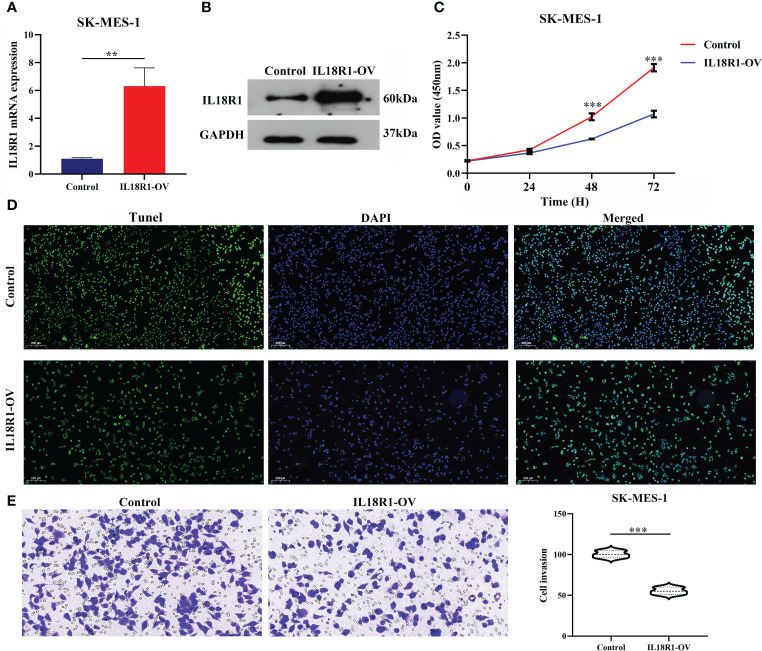
IL18R1 over-expression could inhibit the cell growth and invasion in LUSC. **(A, B)** Validation of cell model using PCR and western blotting; **(C, D)** Cell growth by CCK-8 and Edu methods; **(E)** Cell growth by Transwell method. LUSC, lung squamous cell carcinoma; **P < 0.01; ***P < 0.001.

**Figure 6:**
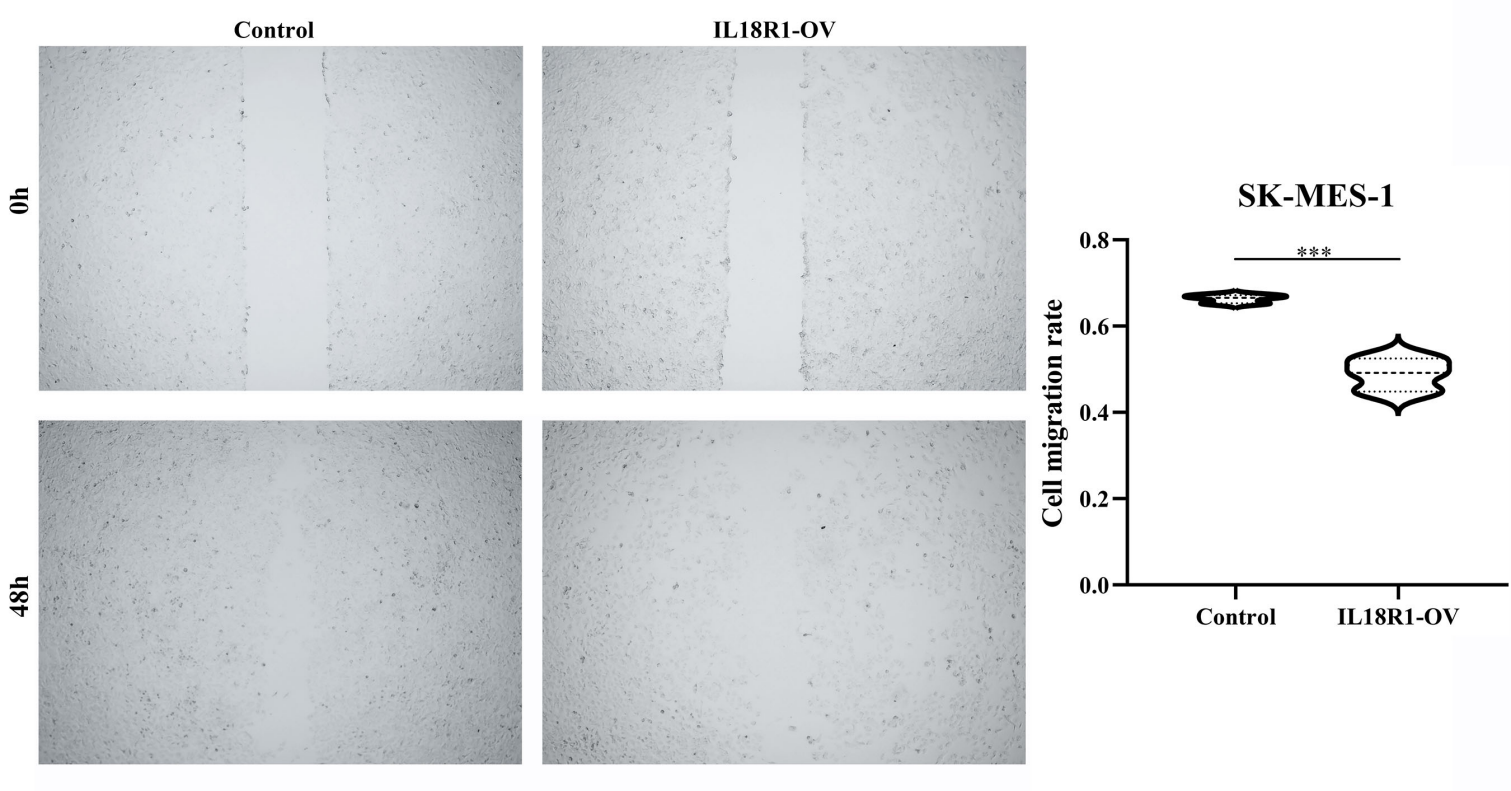
IL18R1 over-expression could inhibit the cell migration in LUSC. LUSC, lung squamous cell carcinoma; ***P < 0.001.

**Figures 5A, B** showed the successful construction of the SK-MES-1 cell model with elevated IL18R1 expression. In our cell model, enhanced IL18R1 expression inhibited the SK-MES-1 cell proliferation, as shown by the CCK-8 method, with statistical significance at 48 and 72 h (**Figure 5C**). Increased IL18R1 expression promoted SK-MES-1 cell apoptosis with a significant difference (**Figure 5D**). In addition, IL18R1 overexpression significantly inhibited the SK-MES-1 cell invasion and migration by scratch and Transwell assays (**Figures 5E**, **6**), suggesting that IL18R1 acts as a tumor suppressor in LUSC progression.

### IL18R1 as a potential biomarker of the LUSC immune microenvironment

**Figure 7:**
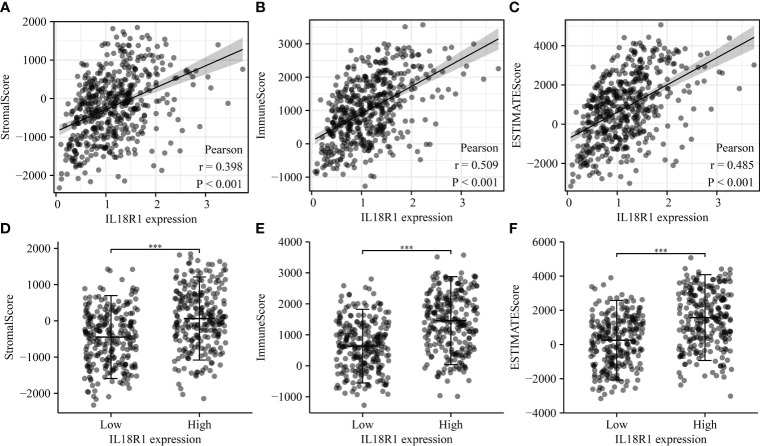
IL18R1 was significantly associated with the levels of stromal, immune, and estimate scores in LUSC. **(A–C)** The relationship between IL18R1 expression and stromal, immune, and estimate scores; **(D–F)** The levels of stromal, immune, and estimate scores in the high and low expression groups of IL18R1. LUSC, lung squamous cell carcinoma.

**Figure 8:**
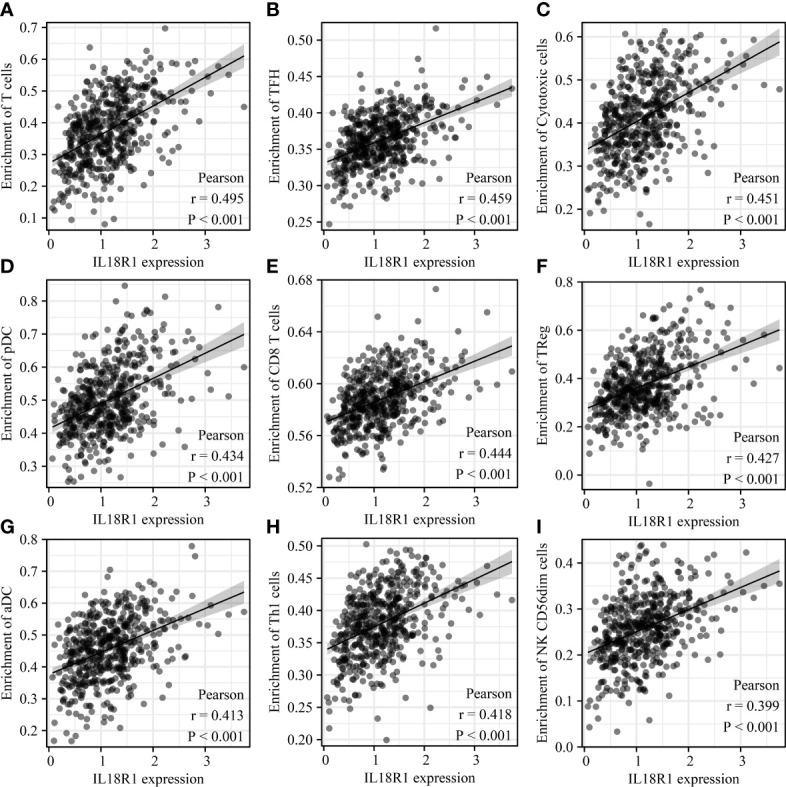
IL18R1 was significantly associated with the levels of immune cells in LUSC. **(A)** T cells; **(B)** TFH; **(C)** Cytotoxic cells; **(D)** pDC; **(E)** CD8 T cells; **(F)** TReg; **(G)** aDC; **(H)** Th1 cells; **(I)** NK CD56dim cells. LUSC, lung squamous cell carcinoma.

The IL18R1 level was significantly relevant to the stromal score (0.398), immune score (0.509) and estimate score (0.485) in LUSC (**Figure 7A–C**). Stromal, immune, and estimate scores showed the significant variation between the high-IL18R1 and low-IL18R1 groups (**Figure 7D–F**). The IL18R1 level was associated with the levels of T cells (0.495), aDC (0.413), cytotoxic cells (0.451), CD8 T cells (0.444), iDC (0.282), pDC (0.434), TReg (0.427), TFH (0.459), Th1 cells (0.418), NK CD56dim cells (0.399), macrophages (0.397), Th2 cells (0.163), T helper cells (0.367), Tem (0.344), B cells (0.363), NK cells (0.363), DC (0.325), eosinophils (0.285), mast cells (0.269), neutrophils (0.239), Tcm (0.191), and Th17 cells (0.122) in LUSC (**Figure 8** and **Table 5**). The T cells, NK CD56dim cells, aDC, mast cells, B cells, eosinophils, NK cells, CD8 T cells, T helper cells, cytotoxic cells, TFH, DC, Th1 cells, pDC, Tcm, neutrophils, Tem, iDC, Tgd, macrophages, and TReg showed significant differences between the high-IL18R1 and low-IL18R1 groups (**Figure S3**).

**Table 5:** IL18R1 was significantly associated with the levels of immune cells in LUSC.

Gene	Immune cells	r	P
IL18R1	T cells	0.495	<0.001
IL18R1	TFH	0.459	<0.001
IL18R1	Cytotoxic cells	0.451	<0.001
IL18R1	CD8 T cells	0.444	<0.001
IL18R1	pDC	0.434	<0.001
IL18R1	TReg	0.427	<0.001
IL18R1	Th1 cells	0.418	<0.001
IL18R1	aDC	0.413	<0.001
IL18R1	NK CD56dim cells	0.399	<0.001
IL18R1	Macrophages	0.397	<0.001
IL18R1	T helper cells	0.367	<0.001
IL18R1	B cells	0.363	<0.001
IL18R1	NK cells	0.363	<0.001
IL18R1	Tem	0.344	<0.001
IL18R1	DC	0.325	<0.001
IL18R1	Eosinophils	0.285	<0.001
IL18R1	iDC	0.282	<0.001
IL18R1	Mast cells	0.269	<0.001
IL18R1	Neutrophils	0.239	<0.001
IL18R1	Tcm	0.191	<0.001
IL18R1	Th2 cells	0.163	<0.001
IL18R1	Th17 cells	0.122	0.006
IL18R1	NK CD56bright cells	-0.033	0.467
IL18R1	Tgd	0.030	0.510

LUSC, lung squamous cell carcinoma.

### Association between IL18R1 level and LUSC immune cell markers

**Figure 9:**
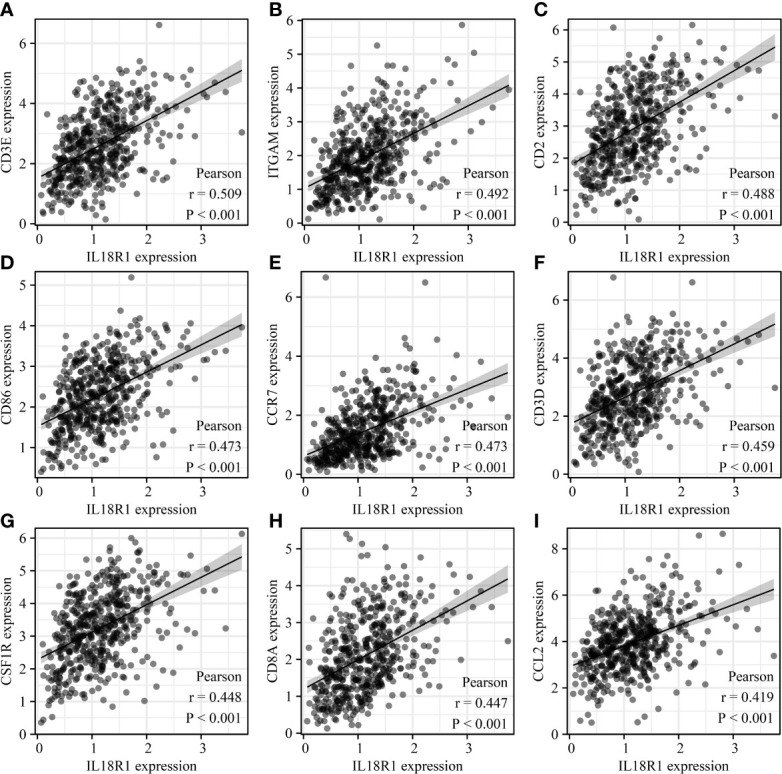
IL18R1 was significantly associated with the levels of immune cell markers in LUSC. **(A)** CD3E; **(B)** ITGAM; **(C)** CD2; **(D)** CD86; **(E)** CCR7;**(F)** CD3D; **(G)** CSF1R; **(H)** CD8A; **(I)** CCL2. LUSC, lung squamous cell carcinoma.

The IL18R1 level were significantly associated the levels of cell markers CTLA4, CD8A, PDCD1, FOXP3, CD8B, KIR2DL4, STAT3, CD2, IL13, CD79A, STAT5A, CSF1R, HAVCR2, TNF, CD68, STAT4, IRF5, CD163, ITGAX, VSIG4, GZMB, CD1C, ITGAM, KIR3DL2, HLA-DRA, KIR2DL1, IFNG, HLA-DPB1, KIR3DL1, HLA-DQB1, LAG3, KIR2DL3, KIR2DS4, HLA-DPA1, CCR7, NRP1, MS4A4A, TBX21, PTGS2, STAT1, IL10, GATA3, CCL2, STAT6, CD86, IL21, CD19, CCR8, CD3E, STAT5B, CD3D, and CEACAM8 in LUSC (**Figure 9** and **Figure S4**).

### IL18R1-related ceRNA network

**Figure 10:**
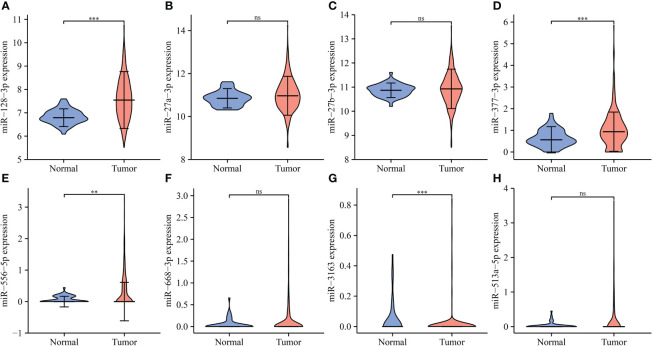
The expression levels of IL18R1-related miRNAs in LUSC. **(A)** miR-128-3p; **(B)** miR-27a-3p; **(C)** miR-27b-3p; **(D)** miR-377-3p; **(E)** miR-556-5p; **(F)** miR-668-3p; **(G)** miR-3163; **(H)** miR-513a-5p. LUSC, lung squamous cell carcinoma; **P < 0.01; ***P < 0.001; ns, no statistical significance.

The miRNAs bound upstream of IL18R1 in the ENCORI database were miR-27a-3p, miR-128-3p, miR-377-3p, miR-513a-5p, miR-27b-3p, miR-556-5p, miR-668-3p, and miR-3163. The data of LUSC patients revealed that miR-128-3p and miR-556-5p were the highly expressed in LUSC tissues and were negatively associated with the IL18R1 expression levels (**Figure 10** and **Figure S5**).

**Figure 11:**
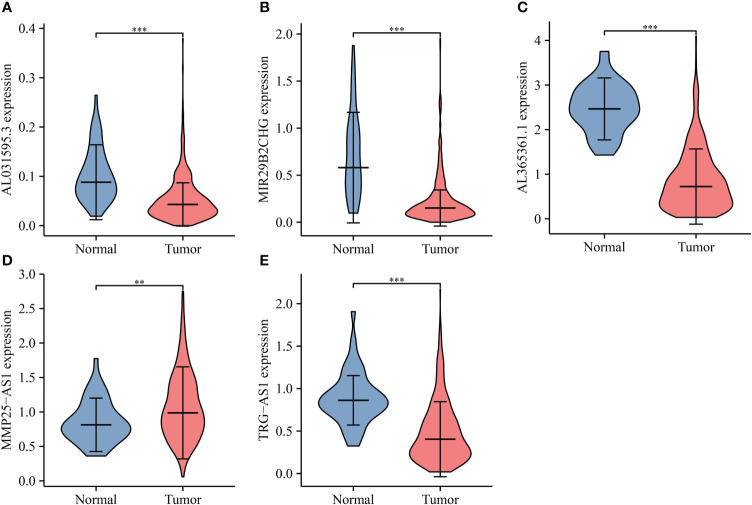
miR-556-5p-related lncRNA expression levels in LUSC. **(A)** AL031595.3; **(B)** MIR29B2CH; **(C)** AL365361.1; **(D)** MMP25-AS1; **(E)** TRG-AS1. LUSC, lung squamous cell carcinoma; **P < 0.01; ***P < 0.001.

**Figure 12:**
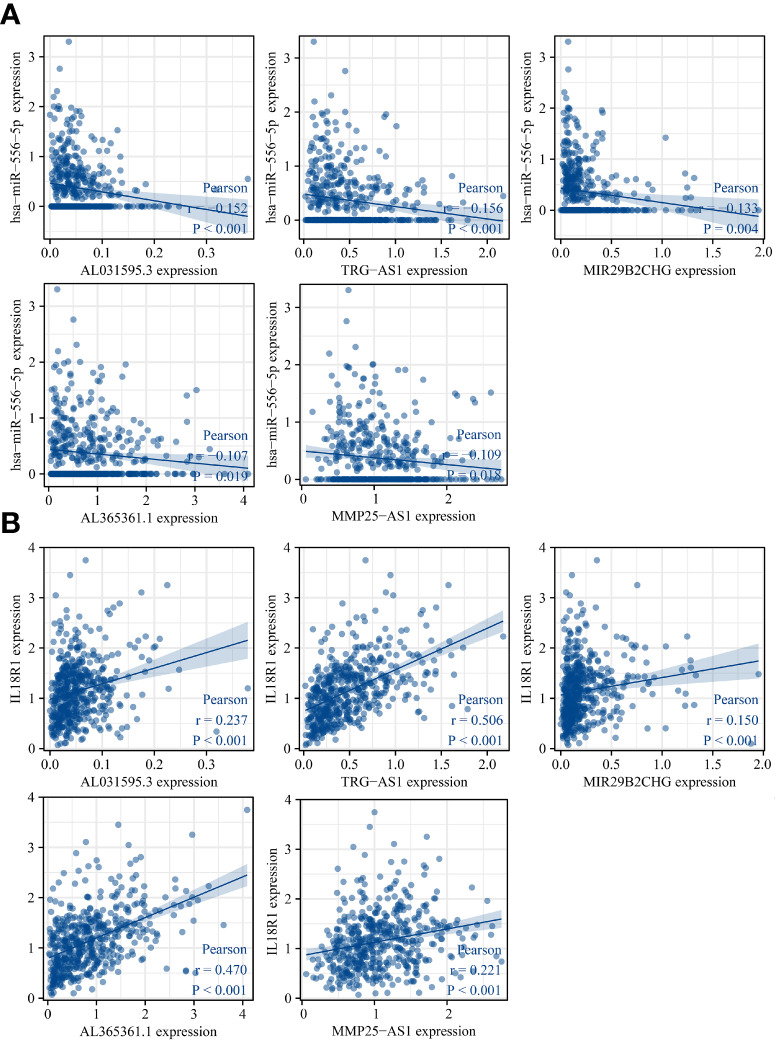
miR-556-5p and IL18R1 expression levels were associated with the AL031595.3, MIR29B2CH, AL365361.1, MMP25-AS1, and TRG-AS1 expression levels in LUSC. **(A)** miR-556-5p; **(B)** IL18R1. LUSC, lung squamous cell carcinoma.

The lncRNAs bound upstream of miR-556-5p in the ENCORI database were the AL365361.1, MIR29B2CHG, TRG-AS1, MMP25-AS1, and AL031595.3. The data of TCGA database revealed that AL365361.1, MIR29B2CHG, TRG-AS1, MMP25-AS1, and AL031595.3 were over-expressed in the LUSC tissues (**Figure 11**), negatively with the miR-556-5p expression level, and positively associated with the IL18R1 expression level (**Figure 12**). However, there was no significant difference between the AL365361.1, MIR29B2CHG, TRG-AS1, MMP25-AS1, and AL031595.3 expression levels and the DSS, OS, and PFI of LUSC patients.

**Figure 13:**
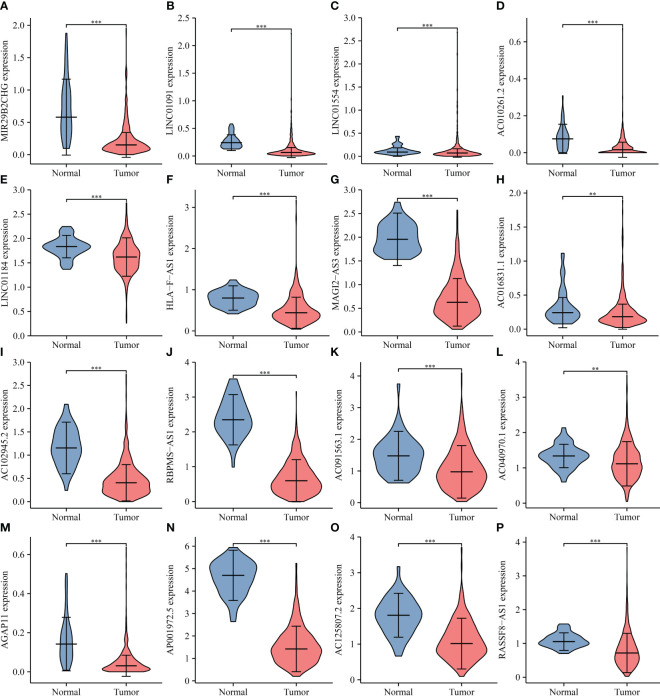
miR-128-3p-related lncRNA expression levels in LUSC. **(A)** MIR29B2CHG; **(B)** LINC01091; **(C)** LINC01554; **(D)** AC010261.2; **(E)** LINC01184; **(F)** HLA-F-AS1; **(G)** MAGI2-AS3; **(H)** AC016831.1; **(I)** AC102945.2; **(J)** RBPMS-AS1; **(K)** AC091563.1; **(L)** AC040970.1; **(M)** AGAP11; **(N)** AP001972.5; **(O)** AC125807.2; **(P)** RASSF8-AS1. LUSC, lung squamous cell carcinoma.

**Figure 14:**
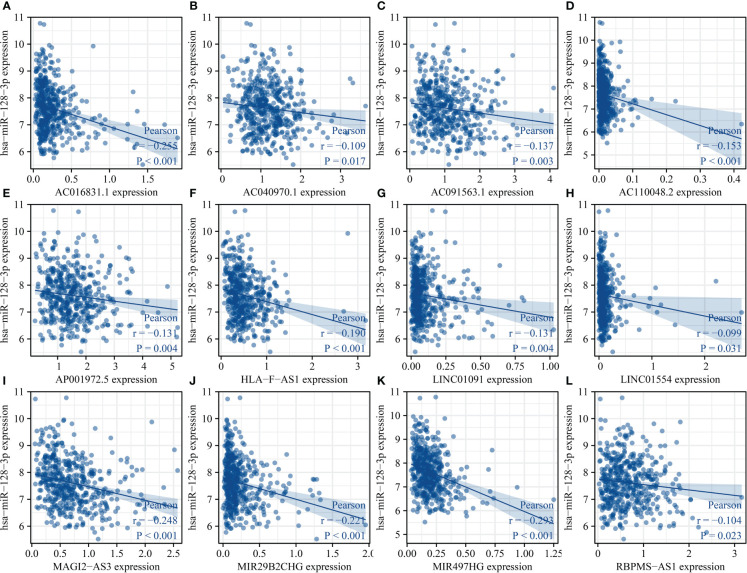
miR-128-3p expression level was associated with the lncRNA expression level in LUSC. **(A)** AC016831.1; **(B)** AC040970.1; **(C)** AC091563.1; **(D)** AC110048.2; **(E)** AP001972.5; **(F)** HLA-F-AS1; **(G)** LINC01091; **(H)** LINC01554; **(I)** MAGI2-AS3; **(J)** MIR29B2CHG; **(K)** MIR497HG; **(L)** RBPMS-AS1. LUSC, lung squamous cell carcinoma.

There were 50 lncRNAs bound of miR-128-3p in the ENCORI database (Table S1). The TCGA database revealed that MIR29B2CHG, LINC01091, LINC01554, AC010261.2, LINC01184, HLA-F-AS1, MAGI2-AS3, AC016831.1, RBPMS-AS1, AC102945.2, AC091563.1, AC040970.1, AGAP11, AP001972.5, AC125807.2, RASSF8-AS1, LINC01550, AC110048.2, MIR497HG, CCDC144NL-AS1, ARHGAP27P1-BPTFP1-KPNA2P3, AC005332.7, AC124319.2, and AL121894.2 were significantly decreased in the LUSC tissues (**Figure 13**). The LINC01091, LINC01554, HLA-F-AS1, MAGI2-AS3, AC016831.1, RBPMS-AS1, AC091563.1, AC040970.1, MIR29B2CHG, AP001972.5, AC110048.2, and MIR497HG expression levels were associated with the miR-128-3p expression (**Figure 14**). Furthermore, the MIR29B2CHG, HLA-F-AS1, MAGI2-AS3, AC016831.1, RBPMS-AS1, AC091563.1, AC040970.1, AP001972.5, AC110048.2, and MIR497HG expression levels were positively associated with the IL18R1 expression level (**Figure S6**). The K-M survival studies demonstrated that decreased RBPMS-AS1, and AC091563.1 expression were related to short DSS or PFI in patients with LUSC, respectively (**Figure S7**), suggesting that IL18R1 competes with RBPMS-AS1 and AC091563.1 to bind miR-128-3p for participating in LUSC progression.

## Discussion

Chemotherapy and immunotherapy of PD-1 can currently increase the chances of surgery in some patients with advanced LUSC for improving the prognosis of patients with LUSC. Studies have confirmed that gene expression changes were involved in the development of LUSC, in turn affecting LUSC progression (18–20). For example, Chen et al. found that PTBP3 was overexpressed in the LUSC tissues. The PTBP3 elevated expression level was associated with dismal prognosis in patients with LUSC. Inhibition of the PTBP3 expression level results in a decrease in the proliferative capacity of the LUSC cells, which could prevent cell cycle progression by downregulating the CDK2/cyclin A2 complex (18). The PCDHA3 expression level was significantly decreased in the LUSC cells. PCDHA3 overexpression reduced the ability of the cell proliferation, invasion, and migration of LUSC. PCHDA3 downregulates the expression of epithelial-mesenchymal transition biomarkers N-cadherin, fibronectin, and vimentin and increases the expression of E-cadherin and α-catenin (19). IL18R1 is a T cytotoxic cell surface marker associated with cancer progression and the immune microenvironment (6–10). Currently, the roles of IL18R1 in LUSC progression and immunity have not been reported. In the present study, we found that IL18R1 was significantly underexpressed in the LUSC tissues. IL18R1 had an AUC of 0.75 in the normal and LUSC tissues, indicating that the IL18R1 expression level plays a role in the diagnosis of LUSC. Survival studies and meta-analysis displayed that decreased IL18R1 level was significantly relevant to dismal prognosis in patients with LUSC. The decreased IL18R1 expression level was an independent risk factor for the OS and DSS in patients with LUSC. Preliminary evidence suggested that IL18R1 might become a prognostic biomarker for patients with LUSC.

In recent years, the ceRNA network is the cancer-related mechanism (21–24). For example, the lncRNA MIR205HG expression level was upregulated in LUSC. Inhibition of MIR205HG expression reduces cancer cell growth, migration, and epithelial-mesenchymal transition processes and promotes cancer cell apoptosis. The decreased MIR205HG expression level could bind to miR-299-3p and promote the upregulation of miR-299-3p expression level, thereby inhibiting the expression of MAP3K2. MIR205HG could accelerate LUSC cell proliferation and progression through the regulation of the miR-299-3p/MAP3K2 mechanism through a ceRNA network (22). The circPVT1 expression level is significantly enhanced in the LUSC tissues. Moreover, the elevated circPVT1 expression level is significantly relevant to shorter OS in patients with LUSC. circPVT1 enhanced the cell proliferation in LUSC and could inhibit the expression of miR-30d and miR-30e through a ceRNA network for promoting CCNF protein expression (24). The expression level of miR-128-3p bound of IL18R1 was significantly increased in the LUSC tissues, and negatively associated with the IL18R1 level. The AC091563.1 and RBPMS-AS1 bound of miR-128-3p expression levels were significantly decreased in the LUSC tissues. The AC091563.1 and RBPMS-AS1 expression levels were negatively relevant to the miR-128-3p expression level, and positively associated with the IL18R1 level. Decreased AC091563.1 level was associated with short DSS in patients with LUSC. Decreased AC091563.1 and RBPMS-AS1 expression levels were associated with short prognosis in patients with LUSC. Moreover, the RBPMS-AS1 and CAMTA1 expression levels were decreased in glioma tissues and cells. RBPMS-AS1 or CAMTA1 overexpression promoted radiosensitivity and cell apoptosis and inhibited cell proliferation in glioma. Furthermore, miR-301a-3p overexpression suppressed radiosensitivity and apoptosis and induced cell proliferation. RBPMS-AS1 or CAMTA1 overexpression could reverse the effects of miR-301a-3p overexpression on glioma cells. To promote cancer progression, RBPMS-AS1 acted as a ceRNA for miR-301a-3p and enhanced the CAMTA1 expression in glioma cells by sponging miR-301a-3p (25). In the cell model, IL18R1 overexpression could inhibit the proliferation, migration, and invasion and promote cell apoptosis of the LUSC cells. It was preliminarily suggested that RBPMS-AS1 competes with IL18R1 to bind miR-128-3p for participating in LUSC progression.

Immunotherapy was considered one of the means of cancer treatment, which could improve the prognosis of patients (26–28). IL18R1 is a T cytotoxic cell surface marker and one of the important molecules in the immune microenvironment (6). In our study, the IL18R1 expression level was significantly associated with the stromal, immune, and estimate scores, and immune cell levels in LUSC. The CD8 T cells, NK CD56dim cells, cytotoxic cells, and other immune cells had significant differences in the high-IL18R1 and low-IL18R1 groups. The IL18R1 expression level was associated with the immune cell markers PDCD1, CTLA4, CD8A, and other markers. Those results indicated that the IL18R1 expression level had a strong association with the LUSC immune microenvironment.

In this study, we discovered the roles of IL18R1 expression in LUSC progression and immunity *via* comprehensive analysis and basic research. Our study has the advantages of large tissue specimens and reliable results. However, more squamous samples were collected to identify the clinical values of IL18R1 and the association between IL18R1 expression and immunity, and the signaling pathways of elevated IL18R1 expression in LUSC progression in model cells need to be explored in the future. Overall, the IL18R1 level was significantly downregulated in the LUSC tissues. Decreased IL18R1 expression played a role in the diagnosis of LUSC and was an independent risk factor for dismal prognosis in LUSC. The IL18R1 level was associated with the LUSC immune microenvironment. AC091563.1 and RBPMS-AS1 could resist the LUSC cell growth and metastasis *via* the miR-128-3p/IL18R1 signaling pathway, indicating that IL18R1 is expected to be a biomarker for evaluating the dismal prognosis of patients with LUSC.

## Data availability statement

The original contributions presented in the study are included in the article/**Supplementary Material**. Further inquiries can be directed to the corresponding authors.

## Ethics statement

The studies involving human participants were reviewed and approved by The ethical committee of Wuhan Union Hospital. The patients/participants provided their written informed consent to participate in this study.

## Author contributions

S-HW, QG, and H-SL designed the theme and research ideas of the article and insisted on the implementation of the plan. QG, C-YW, and NJ wrote the manuscript. ST, J-HW, and H-SL revised our manuscript and improved the language. QG, X-YX, and P-YM downloaded the study data, analyzed the data, and visualized it. NJ and C-YW fed the LUSC cells and performed CCK-8, Tunel, scratch, and Transwell experiments. All authors contributed to the article and approved the submitted version.

## Funding

Our research is supported through the Natural Science Foundation of China (No. 82100115, 82070431, 82100116, and 82100299), and Natural Science Foundation of Hubei (No. 2020CFB392, and 2020CFB818) provided financial support for our research.

## Acknowledgments

We would like to thank the online database websites for freely available data.

## Conflict of interest

The authors declare that the research was conducted in the absence of any commercial or financial relationships that could be construed as a potential conflict of interest.

## Publisher’s note

All claims expressed in this article are solely those of the authors and do not necessarily represent those of their affiliated organizations, or those of the publisher, the editors and the reviewers. Any product that may be evaluated in this article, or claim that may be made by its manufacturer, is not guaranteed or endorsed by the publisher.
