# Supplementary material for: Downregulation of T-cell cytotoxic marker IL18R1 promotes cancer proliferation and migration and is associated with dismal prognosis and immunity in lung squamous cell carcinoma
Source: Front Immunol. 2022 Dec 5;13:986447. doi: 10.3389/fimmu.2022.986447 (PMC9760870; doi:10.3389/fimmu.2022.986447)
Supplement: Supplementary file 1 [file DataSheet_1.docx]

**Supplemental figure legends**


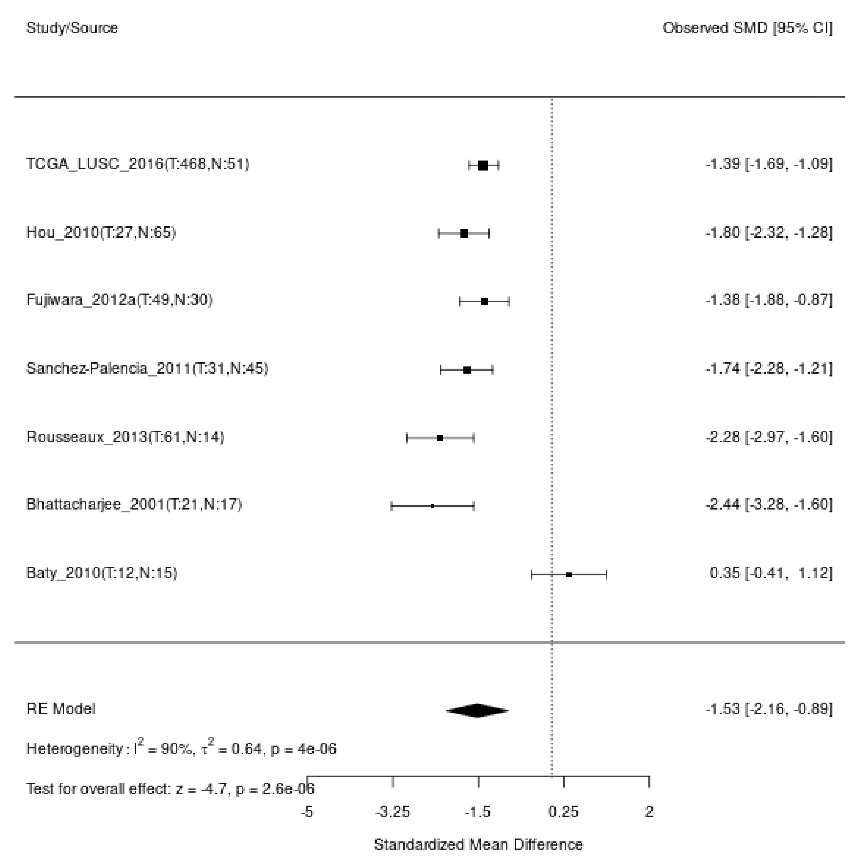


Figure S1. IL18R1 was significantly downregulated in LUSC using meta-analysis.


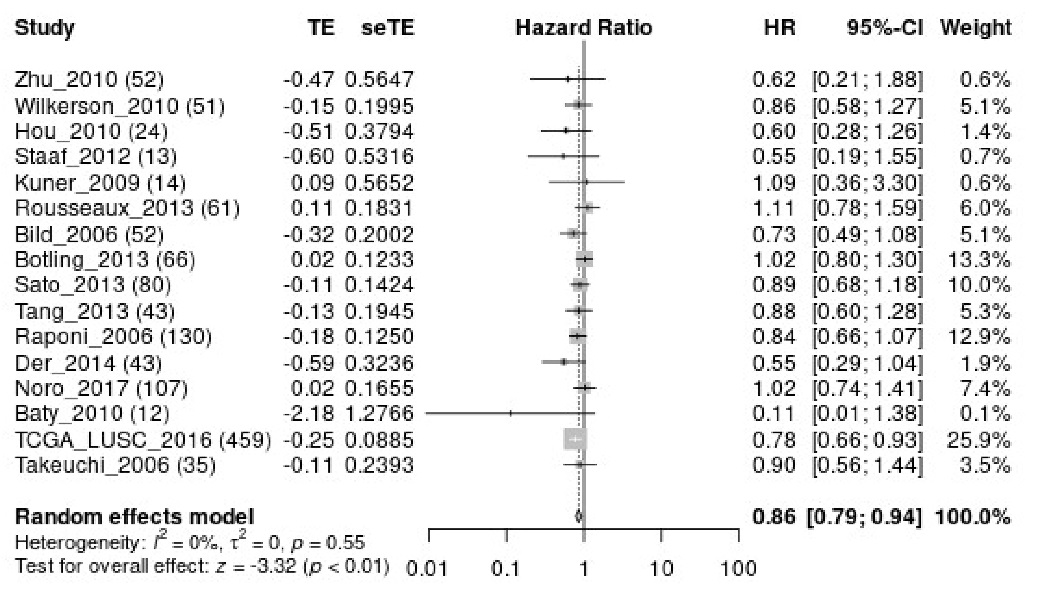


Figure S2. The decreased IL18R1 expression level was significantly associated with dismal prognosis in LUSC using meta-analysis.


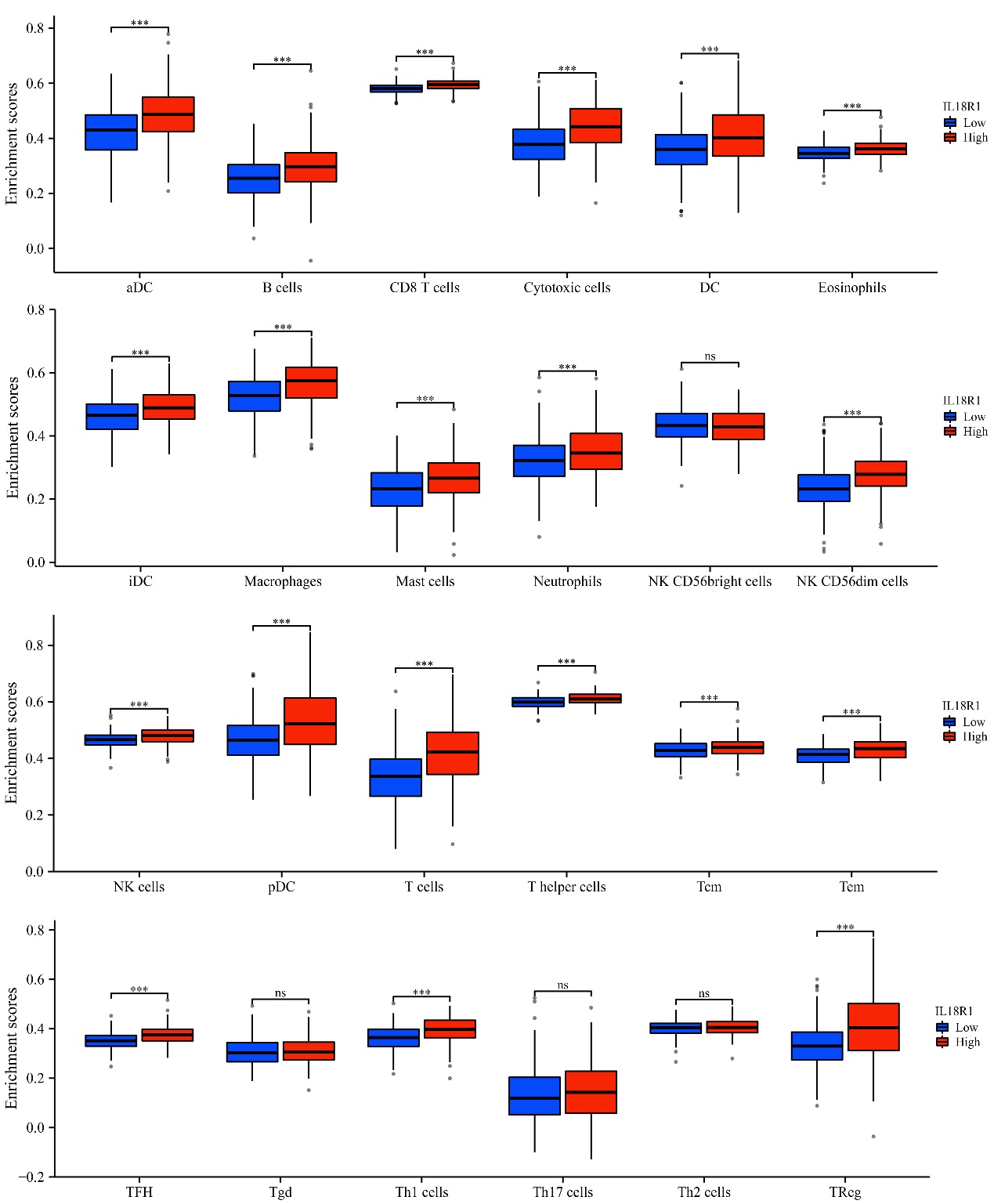


Figure S3. The levels of immune cells in high-IL18R1 and low-IL18R1 expression groups.


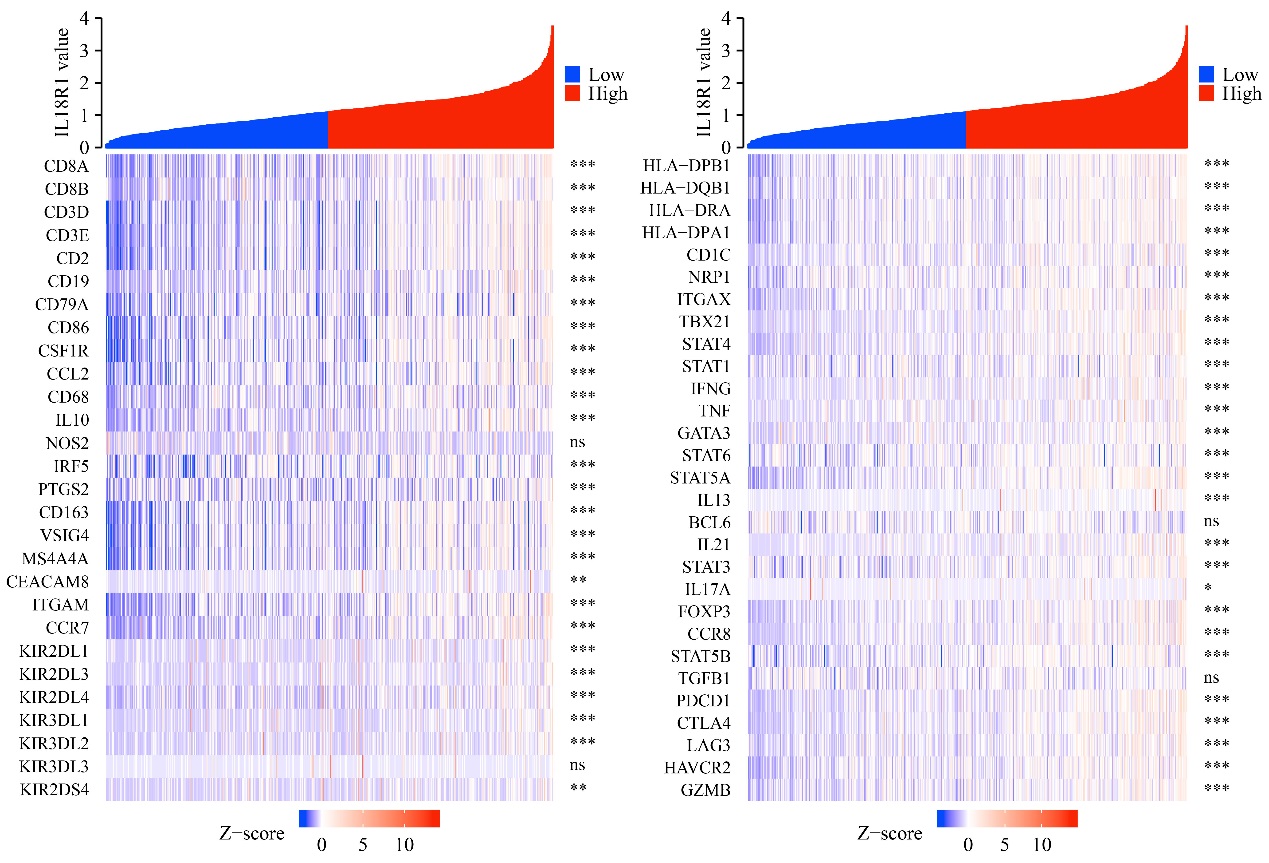


Figure S4. IL18R1 was significantly associated with the levels of immune cell markers in LUSC.

Note: LUSC, lung squamous cell carcinoma.


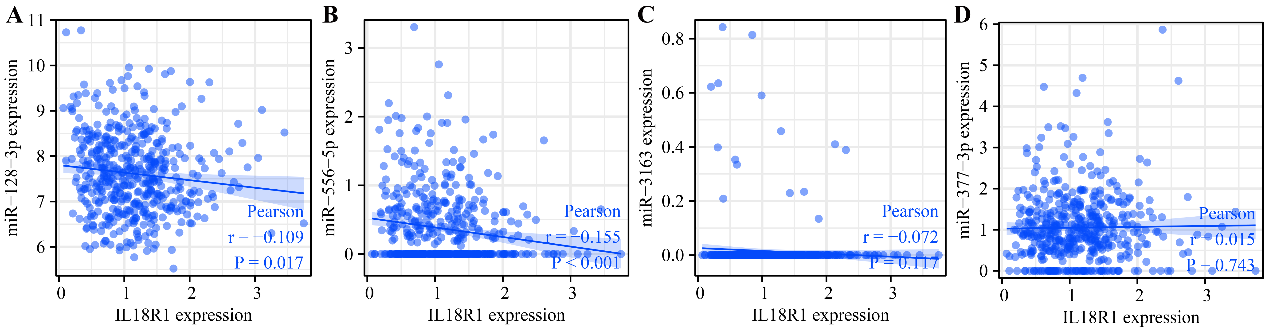


Figure S5. IL18R1 expression was associated with the miRNA expression levels in LUSC. (A) miR-128-3p; (B) miR-556-5p; (C) miR-3163; (D) miR-377-3p.

Note: LUSC, lung squamous cell carcinoma.


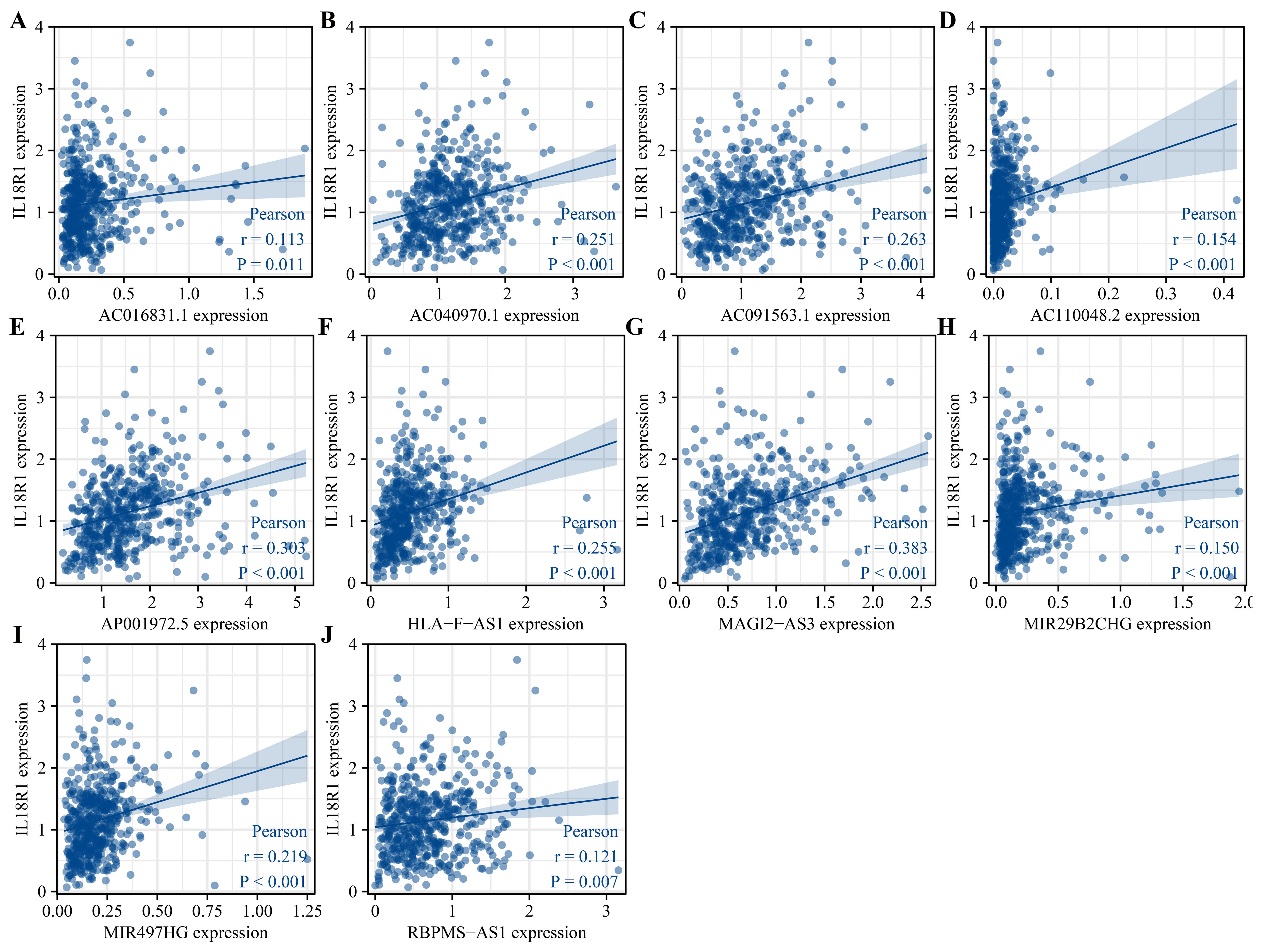


Figure S6. IL18R1 expression levels were associated with the lncRNA expression levels in LUSC.

Note: LUSC, lung squamous cell carcinoma.


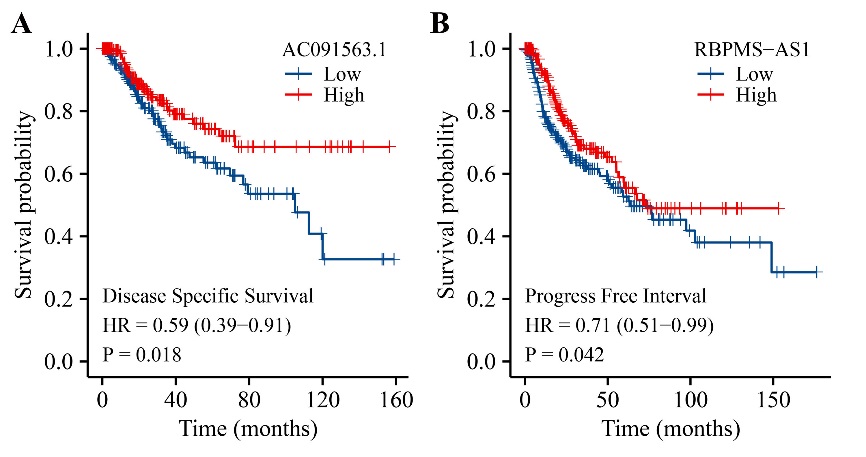


Figure S7. Prognosis-related lncRNAs in LUSC. (A) AC091563.1; (B) RBPMS-AS1.

Note: LUSC, lung squamous cell carcinoma.

Table S1. LncRNAs of miR-128-3p.

| lncRNAs of miR-128-3p | | | | |
| --- | --- | --- | --- | --- |
| AL645608.1 | LINC01554 | AC016831.1 | NEAT1 | AC012181.2 |
| FOXD3-AS1 | AC010261.2 | RBPMS-AS1 | AP001972.5 | AC010533.1 |
| GAS5 | LINC01184 | AC102945.2 | AC021054.1 | MIR497HG |
| MIR29B2CHG | C5orf66 | AC091563.1 | AC125807.2 | CCDC144NL-AS1 |
| CYTOR | AC091825.1 | AC040970.1 | RASSF8-AS1 | ARHGAP27P1-BPTFP1-KPNA2P3 |
| MIR4435-2HG | AC021078.1 | AL158152.1 | AC121338.2 | AC005332.7 |
| LINC01091 | HLA-F-AS1 | ZMIZ1-AS1 | LINC00346 | AC124319.2 |
| AC095055.1 | HCP5 | AGAP11 | LINC01550 | ZNF561-AS1 |
| SCAMP1-AS1 | AC147651.1 | LINC01001 | AC110048.2 | AL118505.1 |
| TMEM161B-AS1 | MAGI2-AS3 | AC240565.2 | AC135050.5 | AL121894.2 |
